# Climate change and pavement burns in the United Kingdom: a case report of two patients

**DOI:** 10.29045/14784726.2023.12.8.3.37

**Published:** 2023-12-01

**Authors:** Emma Whiting, Chiraag Thakrar Karia, Sebastian Tullie, Craig Nightingale, Yvonne Wilson, Alan Kay

**Affiliations:** Queen Elizabeth Hospital Birmingham ORCID iD: https://orcid.org/0000-0003-1212-7474; Queen Elizabeth Hospital Birmingham; Queen Elizabeth Hospital Birmingham; Queen Elizabeth Hospital Birmingham; Queen Elizabeth Hospital Birmingham; Queen Elizabeth Hospital Birmingham

**Keywords:** burns, climate change, United Kingdom

## Abstract

Pavement burns are more common in locations familiarised with high temperatures and a dry climate zone, but have not previously been reported in temperate climates. We present two cases of patients who suffered pavement burns in the United Kingdom during an unprecedentedly hot day in July 2022. The first case involved a 66-year-old male who suffered partial and full thickness burns requiring excision and skin grafting. The second case involved a 58-year-old female with partial thickness burns also requiring excision and skin grafting. Both patients had pre-existing co-morbidities and their pavement burns were precipitated by heat stroke. Pavement burns represent a mechanism of injury that necessitates increased operative management, length of hospital stay and cost per surface area burned when compared to flame or scald burns (Silver et al., 2015). As a result of global warming, we anticipate extreme heat events, and subsequently pavement burns, to increase in incidence in the United Kingdom. There is opportunity for education of the public and health professionals for prevention.

## Introduction

Pavement burns have long been an established risk of direct skin contact with asphalt in high-temperature climates, with the first events (Berens, 1970) and broader aetiological analysis (Harrington et al., 1995) both reported from Arizona-based burn centres. Since then, all reported incidents have occurred in locations known for hot climates, such as Arizona (Harrington et al., 1995), New Mexico (Laarakker et al., 2022) and Israel (Vardy et al., 1989).

Pavement surfaces are often hotter than ambient air temperatures, due to direct sunlight and radiant heat absorption (Laarakker et al., 2022). Surfaces such as asphalt and concrete can become hot enough to cause burns when ambient air temperatures exceed 35°C. For example, in air temperatures of 40°C, unshaded pavement surfaces can reach up to 68.5°C (Clifton et al., 2016), leading to contact burns in less than three seconds (Harrington et al., 1995). Historically, these temperatures have rarely been encountered in the United Kingdom (Met Office, 2022). However, climate change has resulted in the mean UK temperature increasing from 9.14°C in 1991–2020, to a record-breaking 10.03°C in 2022 (Met Office, 2023), along with increased frequency and duration of extreme heat episodes (Kovats & Brisley, 2021). This leads to increased risk of contact pavement burns in the United Kingdom.

We report two cases of contact pavement burns that occurred on the hottest day ever recorded in the United Kingdom (19 July 2022) (Met Office, 2022). The maximum temperature exceeded 40°C in multiple locations across the country. To our knowledge, these are the first reported UK cases of contact pavement burns. This represents a phenomenon that UK-based pre-hospital teams and multidisciplinary burns teams should increasingly be aware of.

## Case 1

**Figure 1:**
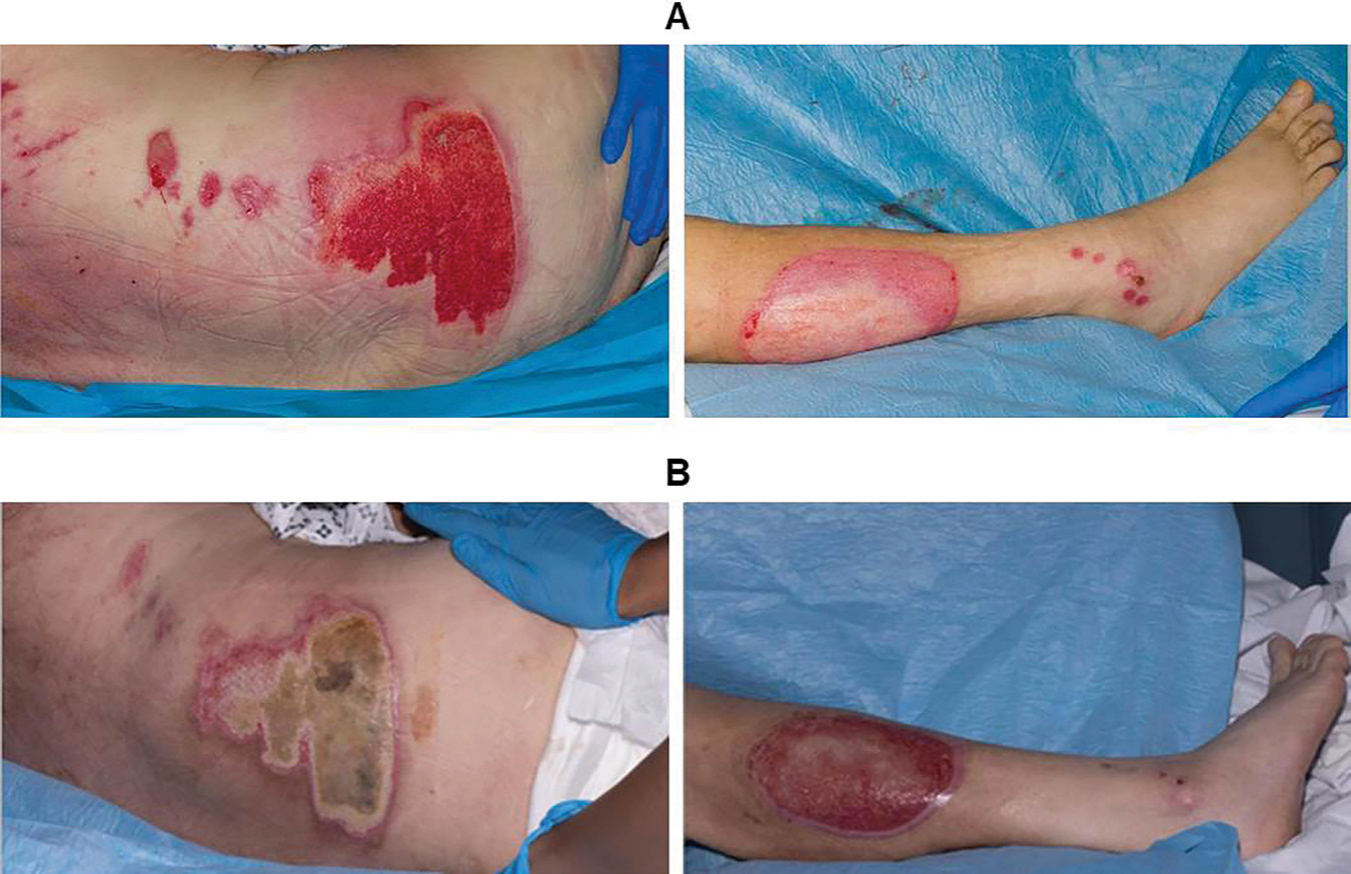
Figure 1. Deep-dermal burn to the right flank and full thickness burn to the right lower leg. A: post-burn day 6; B: post-burn day 20.

A 66-year-old male with a past medical history of Parkinson’s disease, hypertension, type II diabetes and a previous stroke sustained a pavement burn totalling 5% total body surface area (TBSA) (Figure 1). He was found unresponsive while a passenger in a car on 19 July 2022. His family placed him in the recovery position (right lateral recumbent position) on an asphalt car park surface, as advised by the 999 emergency operator. The patient remained on the ground for approximately 15 minutes until an ambulance arrived.

The patient had sustained partial thickness burns to the occiput, right elbow, left elbow and right buttock, a full thickness burn to the right lower leg (1%) and a deep dermal burn to the right flank (1%). He was transferred from his local hospital to the regional burns centre on post-burn day (PBD) 2.

Dressings initially comprised paraffin-impregnated gauze before switching to a daily cerium nitrate-silver sulphadiazine preparation on PBD 3, followed by an anti-microbial foam dressing on PBD 16. Following review by neurology, the cause of his unresponsive episode was attributed to heat stroke and decompensated Parkinson’s disease. Due to the patient’s general frailty, surgical management was initially thought inappropriate. However, his baseline status improved during admission and by PBD 21, the patient was deemed medically fit to undergo an anaesthetic. On PBD 28, excision and split thickness skin grafting to the deeper burn areas of the right flank and right lower leg was performed. A graft check one week post-operatively showed 100% graft take. Other notable events during his in-patient stay included treatment for a urinary tract infection and persistent multi-factorial delirium. The patient was repatriated to his local hospital for ongoing medical care on day 9 post surgery (PBD 37).

## Case 2

**Figure 2:**
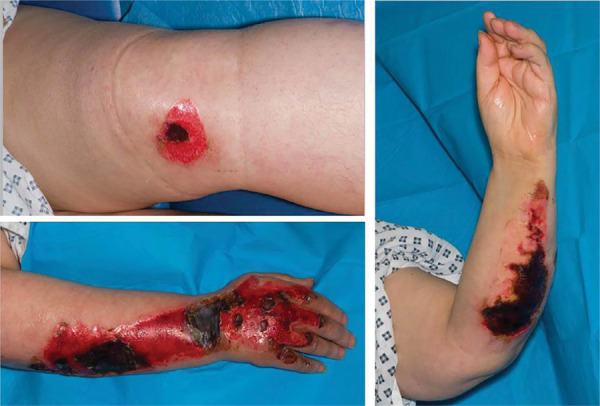
Figure 2. Partial thickness burns to the left knee, right forearm and hand and left forearm.

A 58-year-old female with a past medical history of bipolar disorder, hypertension, hypothyroidism and schizophrenia sustained 2% TBSA partial thickness burns to her bilateral forearms, bilateral hands and left knee (Figure 2). These injuries were sustained after collapsing on pavement near her house following heat stroke. Review of nearby security cameras estimated the length of time on the ground to be 45 minutes. She was transported by ambulance to her local hospital where she was managed for heat stroke and acute kidney injury. The burns were debrided and dressed in paraffin-impregnated gauze during her in-patient stay.

She was discharged from her local hospital on PBD 10, with follow-up at the regional burns centre on PBD 12. On review of the wounds, a decision was made to admit the patient for excision and split thickness skin grafting, which was performed on PBD 15. She remained an in-patient at the regional burns centre until the first graft check on day 4 post surgery (PBD 19), which demonstrated 100% graft take. The patient was discharged on day 7 post surgery (PBD 22), with no other notable in-patient events. She was reviewed once further on day 13 post surgery (PBD 28), when the graft sites had fully healed.

## Discussion

The Earth’s temperature has increased by at least 1.1°C since the last century, with a further expected rise of 0.5°C within the next 20 years (Patel et al., 2022). These climate trends raise the possibility of extreme temperatures (Kovats & Brisley, 2021), and thus pavement burns, in traditionally temperate climates such as the United Kingdom. Compounding this problem is the expansion of urbanised areas, which are prone to the heat island effect. This describes the phenomenon of roads and buildings absorbing and re-emitting heat (Shandas et al., 2019), thus proliferating the potential for pavement burns to occur in urban areas. Of note, both patients discussed were residents in urban areas.

Burn injuries are an important cause of morbidity and mortality worldwide (World Health Organization, 2018). Risk factors for poor outcomes include increasing burn size and depth, very young (< 2 years) or older age (> 60 years) and presence of multiple comorbidities (Huang et al., 2020; Palmieri et al., 2012). Compared to other mechanisms of burn injury, contact pavement burns result in a greater length of hospital stay, a greater need for operative intervention and a greater cost per surface area burned, compared to similarly sized scald or flame burns (Silver et al., 2015). Pavement burns have also been demonstrated to be deeper than suggested by their initial appearance and continue to deepen during a patient’s hospital stay. This is likely due to continued pressure on the wounds while recumbent, as the wounds are often located on pressure points (Silver et al., 2014). Altered mental status, such as that seen with heat stroke, correlates with worse outcomes in patients with pavement burns, including higher 30-day mortality (Berens, 1970). Combined, these factors can lead to a severe, unnecessary burden on hospital services. Awareness of these points could prompt health professionals to have a higher index of suspicion for deeper burn injuries in these patients.

Heat stroke was a pre-disposing factor to the pavement burns in both reported cases. Elderly people and those with co-morbidities are at particular risk of heat stroke due to diminished thermoregulatory capacity (Epstein & Yanovich, 2019). Both patients discussed in this report had significant co-morbidities, which may have contributed to the development of heat stroke and their subsequent injuries. As many cases of heat stroke are preventable (Yeo, 2004), education regarding preventative measures would be beneficial to a UK population previously unaccustomed to such a condition.

There is currently a constraint in the way that pavement burns are coded on the UK National Burn Injury Database. There is no specific option to code an injury as a pavement or asphalt burn, rendering them indistinguishable from other types of contact burn. This needs to be addressed in order to establish seasonal variations and annual trends. Additionally, due to this limitation, it is possible that a series of pavement burns presented to another facility on the same day, which we are unaware of.

There is potential for education of health professionals and the public to prevent contact pavement burns. This is particularly relevant for those administering first aid, during which they may be required to place a patient in the recovery position on a pavement. Further research is needed to determine the optimum approach to these scenarios. However, we suggest that pre-hospital teams and those administering first aid could be alerted to the possibility of pavement burns in hot weather, and precautions taken to avoid direct skin contact of the patient with a pavement. This may include positioning patients in the shade or on a protective surface. The importance of education is also relevant for the parents of young children, who may allow them to venture outside without appropriate protective footwear.

## Conclusion

In conclusion, we have presented the case of two patients who sustained contact pavement burns in the United Kingdom, on a day that exceeded 40°C. The patients required a substantial length of hospital stay, and both underwent operative management of their burns.

Contact pavement burns represent a novel mechanism of injury in the United Kingdom. We expect the incidence of contact pavement burns to increase in the United Kingdom, secondary to climate change. This mechanism of burn injury results in increased length of hospital stay and need for operative intervention compared to scald or flame burns. However, there is scope to educate healthcare professionals and the public on their prevention and management. In particular, ambulance clinicians should be alert for the possibility of pavement burns in patients found on the floor in hot weather.

## Author contributions

All authors contributed to the acquisition of data. EW and CK wrote the first draft of the manuscript. All authors reviewed and edited the manuscript and approved the final version. All authors agree to be accountable for the work, and can identify which co-authors are responsible for other parts of the work. EW acts as the guarantor for this article.

## Conflict of interest

None declared.

## Ethics

Not required. Written consent was obtained for both patients in this case report.

## Funding

None.

## References

[bibr_1] Berens J. J. (1970). Thermal contact burns from streets and highways. *JAMA*, 214(11), 2025–2027. 5536473

[bibr_2] Clifton T. Khoo T. W. Andrawos A. Thomson S., & Greenwood J. E. (2016). Variation of surface temperatures of different ground materials on hot days: Burn risk for the neuropathic foot. *Burns*, 42(2), 453–456. 26797153 10.1016/j.burns.2015.08.026

[bibr_3] Epstein Y., & Yanovich R. (2019). Heatstroke. *The New England Journal of Medicine*, 380(25), 2449–2459. 31216400 10.1056/NEJMra1810762

[bibr_4] Harrington W. Z. Strohschein B. L. Reedy D. Harrington J. E., & Schiller W. R. (1995). Pavement temperature and burns: Streets of Fire. *Annals of Emergency Medicine*, 26(5), 563–568. 7486363 10.1016/s0196-0644(95)70005-6

[bibr_5] Huang Y.-Z. Lu G.-Z. Zhao H.-S. Liu L.-J. Jin J. Wu Y.-F. Wu J. Zhao F.-L. Liu N. Liu W.-M. Liu L. Zhu T.-J. Chen E.-Z. Gu Q. Ye H.-W. Xi X.-M. Du B. Yi Y., & Qiu H.-B. (2020). Clinical features and mortality-related factors of extensive burns among young adults: The Kunshan disaster experience. *Annals of Translational Medicine*, 8(17), 1053. 33145272 10.21037/atm-20-288 PMC7575965

[bibr_6] Kovats S., & Brisley R. (2021). Health, communities and the built environment. In R. A. Betts A. B. Haward, & K. V. Pearson (Eds.), *The third UK climate change risk assessment technical report*. Prepared for the Climate Change Committee, London. https://www.ukclimaterisk.org/wp-content/uploads/2021/06/CCRA3-Chapter-5-FINAL.pdf.

[bibr_7] Laarakker A. S. Rich A., & Wu E. (2022). Pavement burns in New Mexico: Our experiences, treatments, and outcomes. *Journal of Burn Care and Research*, 43(1), 281–286. 34358305 10.1093/jbcr/irab154

[bibr_8] Met Office. (2022). *A milestone in UK climate history*. https://www.metoffice.gov.uk/about-us/press-office/news/weather-and-climate/2022/july-heat-review.

[bibr_9] Met Office. (2023). *Climate change drives UK’s first year over 10°C*. https://www.metoffice.gov.uk/about-us/press-office/news/weather-and-climate/2023/climate-change-drives-uks-first-year-over-10c.

[bibr_10] Palmieri T. L. Molitor F. Chan G. Phelan E. Shier B. J. Sen S., & Greenhalgh D. G. (2012). Long-term functional outcomes in the elderly after burn injury. *Journal of Burn Care and Research*, 33, 497–503. 22777398 10.1097/BCR.0b013e31825aeaac

[bibr_11] Patel L. Conlon K. C. Sorensen C. McEachin S. Nadeau K. Kakkad K., & Kizer K. W. (2022). Climate change and extreme heat events: How health systems should prepare. *NEJM Catalyst: Innovations in Care Delivery*, 3(7). https://doi.org/10.1056/CAT.21.0454.

[bibr_12] Shandas V. Voelkel J. Williams J., & Hoffman J. (2019). Integrating satellite and ground measurements for predicting locations of extreme urban heat. *Climate*, 7(1), 5. https://doi.org/10.3390/CLI7010005.

[bibr_13] Silver A. G. Dunford G. M. Zamboni W. A., & Baynosa R. C. (2015). Acute pavement burns: A unique subset of burn injuries: A five-year review of resource use and cost impact. *Journal of Burn Care and Research*, 36(1), e7–e11. 25207798 10.1097/BCR.0000000000000162

[bibr_14] Silver A. G. Zamboni W. A., & Baynosa R. C. (2014). Operative management of acute pavement burns: A case series. *Journal of Wound Care*, 23(11), 563–569. 25375404 10.12968/jowc.2014.23.11.563

[bibr_15] Vardy D. A. Khoury M. Ben-Meir P. Ben-Yakar Y., & Shoenfeld Y. (1989). Full skin thickness burns caused by contact with the pavement in a heat-stroke victim. *Burns*, 15(2), 115–116. 2736047 10.1016/0305-4179(89)90141-1

[bibr_16] World Health Organization. (2018). *Burns*. https://www.who.int/news-room/fact-sheets/detail/burns.

[bibr_17] Yeo T. P. (2004). Heat stroke: A comprehensive review. *AACN Clinical Issues*, 15(2), 280–293. 15461044 10.1097/00044067-200404000-00013

